# Dietary strategies for chronic constipation: smartly targeting hormonal and reflex pathways for optimal recovery

**DOI:** 10.3389/fphar.2026.1738562

**Published:** 2026-02-18

**Authors:** Emanuela Ribichini, Giulia Scalese, Chiara Mocci, Natascia De Amicis, Carola Severi

**Affiliations:** Department of Translational and Precision Medicine, Sapienza University of Rome, Rome, Italy

**Keywords:** chronic constipation, chrono-nutritional timing, enteroendocrine signalling, gastrocolic reflex, gut brain axis, personalized nutrition

## Abstract

Chronic constipation (CC) is a common disorder of gut-brain interaction that markedly impairs quality of life and remains challenging to manage. Despite the availability of laxatives and prosecretory agents, up to half of patients experience suboptimal relief, underscoring the need for complementary, physiology-based nutritional strategies. Nutrients influence intestinal motility through multiple pathways, including enteroendocrine signalling, bile-acid metabolism, microbiota-derived metabolites, and intestinal taste-receptor activation. Integrating these mechanisms into clinical nutrition requires structured approaches that align fiber type, bile flow, microbial modulation, and sensory stimulation with motility phenotype and fermentation tolerance. Among dietary interventions, the most consistent clinical evidence supports the use of soluble fiber (e.g., psyllium), kiwifruit or prunes, and magnesium- or sulfate-rich mineral waters to improve stool frequency and consistency. Other components, such as fermented foods, probiotics, and generic hydration, show variable efficacy and remain supported primarily by physiological or translational data. The SMART (Sensory, Motor, bile Acid and Reflex Tailored) Constipation Diet (SCD) is proposed as a hypothesis-generating dietary framework that integrates fiber optimization, bile stimulation, microbial support, and chrono-nutritional timing into a coherent dietary model. Given the heterogeneity of CC (e.g., functional constipation, IBS-C, defecatory disorders) and the scarcity of phenotype-stratified trials, the SCD should be regarded as a translational concept rather than a validated clinical protocol. Future randomized, controlled studies with hard motility and symptom outcomes are needed to determine whether coordinated, multi-pathway dietary modulation can outperform single-component interventions and advance precision nutrition in CC.

## 1 Introduction

Chronic constipation (CC) is classified among the disorders of gut–brain interaction (DGBIs), a group of functional gastrointestinal (GI) conditions in which altered communication between the enteric and central nervous systems contributes to symptom generation (Drossman and Hasler, 2016).

Clinically, CC is defined by persistently difficult or infrequent defecation, excessive straining, hard or lumpy stools, and a sensation of incomplete evacuation. According to Rome IV criteria, symptoms must be present for at least 3 months, with onset at least 6 months prior to diagnosis (Sperber et al., 2021). Within this framework, CC encompasses three main entities with partially distinct pathophysiological substrates relevant to dietary modulation. Functional constipation is predominantly characterized by impaired colonic propulsion and reduced stool hydration, often associated with delayed transit and diminished postprandial motor responses (Lembo, 2016). Constipation-predominant irritable bowel syndrome (IBS-C) shares features of altered motility but is additionally marked by visceral hypersensitivity, dysregulated enteroendocrine signaling, and enhanced gut-brain interactions, which may influence tolerance to specific nutrients and fermentable substrates (Ford et al., 2017; Staudacher et al., 2014; Whelan et al., 2024). Functional defecatory disorders are defined by abnormal recto-anal coordination and pelvic floor dyssynergia during attempted defecation, with relatively preserved colonic transit but ineffective expulsion, limiting the efficacy of dietary interventions alone (Rao and Patcharatrakul, 2016).

Physiological and transit studies highlight the marked heterogeneity of CC, which may present with normal or delayed colonic transit and with different motor patterns, ranging from reduced propulsive activity to increased segmental tone. Although these motor patterns do not represent a formal classification, they provide a useful pathophysiological context to interpret symptom heterogeneity and potential differential responses to dietary and behavioral interventions (Lembo, 2016; Sadeghi et al., 2023).

In addition, it is possible to make a descriptive, non-classificative distinction between hypotonic and hypertonic motor patterns, identified in physiological and manometric studies. Hypotonic patterns are characterized by reduced propulsive activity and blunted postprandial responses, whereas hypertonic patterns are associated with increased non-propulsive segmental contractions and elevated colonic tone (Bassotti et al., 2005; Ravi et al., 2010; Cuomo and Habib, 2007). Although not incorporated into current classification systems, these functional differences may help explain symptom heterogeneity and, on a speculative basis, provide a mechanistic rationale for differential dietary approaches, pending validation in controlled clinical studies.

In this complex umbrella of phenotypes, a step-up approach, starting from shared therapeutic strategies and progressing toward personalized interventions, has been proposed. The first level includes lifestyle and dietary measures, followed by osmotic or stimulant laxatives and, when needed, prokinetic or secretagogue agents or transanal irrigation (Corsetti et al., 2021).

Dietary modification is universally recommended as a first-line or adjunctive measure, particularly in functional constipation (Bharucha and Lacy, 2020). However, current guidelines do not specify for which clinical severity or phenotype dietary interventions are sufficient as stand-alone therapy, or how they should be integrated with laxatives in moderate-to-severe disease.

This lack of stratification has led to the widespread application of uniform dietary advice across heterogeneous patient populations. Clarifying the scope and limits of dietary therapy, and its potential synergy with pharmacologic treatments, remains a critical unmet need. Existing nutritional guidance largely derives from studies testing isolated components, soluble and insoluble fibers, specific fruits, or mineral waters, rather than structured dietary models targeting physiological mechanisms (Dimidi et al., 2014).

Few studies have systematically explored how nutrients and meal patterns modulate enteroendocrine signalling, bile flow, and postprandial reflexes that collectively regulate GI motility. This perspective moves beyond stool bulking and osmotic mechanisms toward neurohormonal and reflex regulation of motility, encompassing the roles of gut hormones, bile acids (BAs), and the gastrocolic reflex (Camilleri, 2019).

What distinguishes this review is its integrative focus on hormonal and reflex pathways as explicit dietary targets. We synthesize current evidence on how nutrients and meal patterns influence gut-hormone secretion, bile-acid signalling, and microbial metabolites to modulate intestinal transit and symptom expression in CC. Rather than viewing diet solely as mechanical or osmotic support, we propose it as a tool for neurohormonal and reflex modulation, potentially enabling individualized, physiology-based strategies.

To ensure scientific rigor, the review distinguishes three evidence layers: (i) physiology (animal or healthy-volunteer data), (ii) translational signals (biomarker or pilot studies), and (iii) clinical evidence (controlled trials in CC). Where data are limited, statements are presented as testable hypotheses rather than prescriptive recommendations.

In this context, we introduce the SMART Constipation Diet (SCD), a Sensory, Motor, Acid (bile acid), and Reflex-Oriented dietary as a translational, hypothesis-generating framework that integrates mechanistic principles into practical nutritional guidance. Given the phenotypic heterogeneity of CC (slow-transit, IBS-C, defecatory disorders), dietary responses are expected to vary by motility pattern, fermentation tolerance, and symptom severity, and should be aligned with pharmacologic and behavioral therapies in more severe presentations (Bharucha et al., 2013).

## 2 Physiological basis of diet-motility modulation

The physiological control of intestinal motility relies on the coordinated action of neural, hormonal, nutrient sensing, and microbial pathways. Each of these systems is profoundly influenced by the nutritional environment. Luminal nutrients are detected by chemosensory and mechanosensory receptors located on enteroendocrine and enteric neurons, which translate food-derived stimuli into integrated neurohormonal responses regulating transit, secretion, and satiety.

Intestinal motility is governed by the integrated activity of the enteric, autonomic, and central nervous systems, which coordinate propulsion, secretion, and absorption (Grundy and Schemann, 2006). The enteric nervous system (ENS) consists of two main plexuses: the myenteric plexus (Auerbach) that regulates smooth muscle tone and peristalsis, and the submucosal plexus (Meissner) that controls secretion and mucosal blood flow (Costa and Brookes, 1994).

Cholinergic excitatory neurons release acetylcholine to stimulate muscle contraction, while inhibitory motor neurons release nitric oxide and vasoactive intestinal peptide (VIP) to relax downstream segments (Furness, 2012). Both types of neurons are activated by serotonin (5-hydroxytryptamine, 5-HT) released by enterochromaffin cells in response to luminal contents (Mawe and Hoffman, 2013).

Ascending and descending pathways connect the ENS with the brainstem and spinal cord, forming the neural substrate of the gut–brain axis (GBA) that integrates autonomic and emotional inputs with intestinal activity (Mayer et al., 2015). Parasympathetic (vagal and pelvic) efferents enhance motility and secretion, whereas sympathetic fibers exert noradrenergic inhibition and increase sphincter tone (Sarna, 1991).

Disruption of these networks, through neuropathy, inflammation, or stress-related dysregulation, impairs coordination of contraction and relaxation, predisposing to motility disorders such as CC.

### 2.1 Reflex pathways

Reflex pathways coordinate regional motor activity ensuring synchronization of motility among different GI regions. Three major classes of reflexes are recognized, classified by their neural circuitry and degree of central involvement (Bharucha et al., 2006).

Intrinsic reflexes, confined within the ENS, mediate local motor patterns such as peristalsis and segmentation through balanced activation of excitatory cholinergic and inhibitory nitrergic neurons (Furness, 2012; Grundy and Schemann, 2006).

Prevertebral reflexes, transmitted via sympathetic ganglia, integrate activity between distant gut regions. The gastrocolic reflex, triggered by gastric distension, enhances colonic motility; the enterogastric reflex inhibits gastric emptying in response to duodenal stimuli; and the coloileal reflex delays ileal flow during colonic distension to prevent downstream overload (Sarna, 1991).

Centrally integrated reflexes involve vagovagal circuits in the brainstem and spinal pathways coordinating swallowing, gastric accommodation, and defecation, which requires coordinated colonic and rectal contraction with pelvic floor relaxation (Bharucha et al., 2006; Sadeghi et al., 2023).

Together, these hierarchical reflex networks maintain efficient propulsion and reservoir functions that need to be strictly regulated to avoid dismotility (Bharucha et al., 2013).

### 2.2 Nutrient sensing and neurohormonal regulation of gut motility

Meal ingestion is a major physiological trigger of colonic motility, activating the gastrocolic reflex and increasing colonic tone and propagated contractions through integrated neural, hormonal, and bile-acid-dependent mechanisms, with maximal responses typically occurring after breakfast (Rao et al., 2000; Tack et al., 2006; Camilleri, 2019). In CC, particularly in slow-transit forms, postprandial motor responses are often blunted or disorganized, contributing to impaired propulsion (Bassotti et al., 2005; Rao and Sun, 1997). Gastrointestinal motility is finely tuned by a network of enteroendocrine peptides that couple luminal nutrient sensing with neural and muscular responses (Daly et al., 2013). These hormones act through specific receptors on enteric neurons and smooth muscle, forming a feedback system that integrates nutritional state, luminal composition, and autonomic tone (Furness, 2012; Janssen et al., 2011). Dysregulation of this neurohormonal axis, as observed in CC, alters the balance between propulsive and inhibitory signalling, leading to delayed colonic transit and impaired evacuation (Young et al., 2013).

During fasting, cyclic release of motilin initiates the migrating motor complex (MMC), clearing the upper intestine before subsequent meals (Ohno et al., 2010). Shortly before eating, ghrelin secreted by gastric X/A-like cells stimulates gastric contractions and accelerates emptying (Tack J et al., 2006). Following food intake, several peptides transiently slow proximal propulsion to allow effective mixing of luminal contents with BAs and digestive enzymes, optimizing digestion and nutrient absorption. Cholecystokinin (CCK) and peptide YY (PYY) are secreted in proportion to the fat and protein content of meals, delaying gastric emptying and small-intestinal transit while coordinating gallbladder contraction and pancreatic secretion (Beglinger and Degen, 2006; Batterham et al., 2002; Drucker, 2006). The incretin glucagon-like peptide-1 (GLP-1), secreted by ileal L cells, also inhibits antral and small-intestinal motility (Drucker, 2006; Nauck and Meier, 2018).

Nutrient sensing is further mediated by chemosensory receptors expressed on enteroendocrine cells (EECs), brush cells, and afferent neurons, primarily belonging to the taste receptor type 1 (TAS1R) and type 2 (TAS2R) families (Depoortere, 2014). These receptors translate the chemical profile of ingested food into hormonal and neural signals. Sweet (TAS1R2-TAS1R3) and umami (TAS1R1-TAS1R3) receptors respond to carbohydrates and amino acids, respectively, whereas bitter TAS2Rs detect plant-derived compounds and BAs, fine-tuning the secretion of CCK, GLP-1, and PYY (Daly et al., 2013; Janssen et al., 2011). Moderate activation of sweet and umami receptors promotes physiological postprandial peristalsis, whereas chronic overstimulation by high-sugar diets may blunt receptor sensitivity, impairing reflex motility and favoring dyspeptic or constipated phenotypes. Conversely, stimulation of TAS2R receptors by bitter compounds or BAs can exert dual effects, inhibiting gastric accommodation proximally while enhancing CCK-mediated propulsive activity distally (Depoortere, 2014; Chen et al., 2006) providing a mechanistic basis for the traditional use of bitter foods to stimulate bowel function.

Intestinal sweet and bitter taste receptors are expressed throughout the GI tract, where they modulate hormone secretion and motility. Although altered TAS1R/TAS2R signalling has been described mainly in metabolic conditions, evidence in disorders of gut-brain interaction remains limited and largely physiological (Young et al., 2013). In the context of CC, reduced chemosensory responsiveness may attenuate enteroendocrine stimulation required for efficient reflex activation, representing a plausible sensory contribution to hypomotility.

Finally, BAs act as potent signalling molecules that link lipid digestion to motor control through nuclear and membrane receptors (Lefebvre et al., 2009). FXR-FGF19 signalling exerts inhibitory effects on motility, whereas TGR5 activation on enteric neurons and epithelial cells enhances chloride secretion, smooth-muscle relaxation, colonic propulsion, and neuromodulation (Poole et al., 2010; Wahlström et al., 2016). Impaired bile-acid metabolism or reduced fecal BA excretion, as observed in slow-transit CC, provides a mechanistic rationale for dietary and therapeutic strategies aimed at enhancing bile flow or receptor activation (Camilleri, 2015; Vijayvargiya et al., 2018).

### 2.3 The gut microbiota and its metabolites as mediators of motility

The gut microbiota functions as a metabolic and neuroendocrine organ, translating dietary composition into signals that regulate intestinal motility (Yano et al., 2015; Wei et al., 2022). The products of microbial fermentation exert distinct effects on gut motor activity. Methane, mainly produced by Methanobrevibacter smithii, increases smooth-muscle tone and slows transit, contributing to slow-transit constipation (Pimentel et al., 2006). Hydrogen sulphide (H_2_S), another gaseous metabolite, acts as a signalling molecule with biphasic effects: low concentrations promote relaxation, whereas higher levels inhibit propulsion, indicating a complex modulatory role in motility control (Jimenez et al., 2017).

In methane-positive or bloating-prone individuals, targeted suppression of methanogens with antibiotics or dietary modulation of fermentable substrates has been shown to improve transit time (Rezaie et al., 2017; Ghoshal et al., 2018).

Short-chain fatty acids (SCFAs), the main products of gut microbiota fermentation of soluble fibers, activate free fatty acid receptors (FFAR2 and FFAR3) on enteroendocrine L cells, stimulating GLP-1 and PYY secretion and modulating motility (Chambers et al., 2018). In parallel, SCFAs enhance mucosal serotonin release, reinforcing peristaltic reflexes through enteric signalling (Yano et al., 2015).

Secondary BAs (deoxycholic and lithocholic acids), other microbial-derived metabolic products generated by bacterial dehydroxylation, enhance propulsive activity and stimulate colonic secretion (Ridlon et al., 2016). Similarly, tryptamine, derived from bacterial tryptophan metabolism, activates mucosal 5-HT4 receptors, enhancing peristaltic reflexes (Bhattarai et al., 2018).

Altered microbial composition or reduced fermentation capacity can impair this endocrine-neural interface of motility control. Overall, microbial patterns enriched in mucin-degrading taxa and their predicted metabolites, such as N-acetyl-D-glucosamine, have been associated with delayed colonic transit, suggesting a potential mucin-epithelium-motility axis relevant to slow-transit phenotypes (Wu et al., 2024).

## 3 Nutrient modulation and dietary strategies in chronic constipation

Each macronutrient, carbohydrate, fat, protein, and fiber, activates distinct sensory and signalling cascades that converge to fine-tune intestinal propulsion (Voigt et al., 2019). Through this integrated network, diet functions not merely as an energy source but as a central regulator of motility and intestinal homeostasis. Moreover, macronutrient quality and palatability influence postprandial motor responses via taste-receptor activation (Depoortere, 2014).

This chapter examines how specific nutrients and dietary patterns engage these regulatory pathways, highlighting mechanistic and translational insights that inform dietary strategies for CC, while distinguishing clinically supported interventions from those that remain hypothesis-generating and require validation in phenotype-stratified studies.

Dietary fiber is the most extensively studied nutritional determinant of intestinal motility, acting through mechanical, endocrine, and microbial mechanisms that influence transit and stool consistency (Anderson et al., 2009). Beyond simple bulking, fiber modulates neurohormonal and microbial pathways that connect luminal fermentation with enteric signalling and colonic reflexes. Soluble fibers, notably psyllium, inulin, β-glucans, and partially hydrolyzed guar gum (PHGG), are moderately fermentable substrates for Bifidobacterium and *Lactobacillus*, generating SCFAs such as acetate, propionate, and butyrate (Yang et al., 2012). SCFAs activate FFAR2/3 receptors and stimulate GLP-1, PYY, and 5-HT release, reinforcing propulsive motor patterns (Chambers et al., 2018).

Clinical evidence consistently supports psyllium as the most effective fiber supplement in CC: randomized trials using 6–12 g/day report improved stool frequency, water content, and ease of passage, often reducing laxative need (Yang et al., 2012). In contrast, insoluble fibers such as wheat bran primarily increase fecal bulk through mechanical distension but show variable efficacy and can exacerbate bloating or discomfort in IBS-C or methane-positive phenotypes (Badiali et al., 1995). As highlighted by Müller-Lissner et al. (2005), constipation is not uniformly fiber-deficient, and excessive insoluble fiber may worsen symptoms in selected patients.

From a translational perspective, the efficacy of fiber depends on fermentability, solubility, and adaptation of the microbiota. A gradual increase to 25–30 g/day of total fiber, distributed across meals and accompanied by adequate hydration (1.5–2 L/day), optimizes tolerance and fermentation kinetics (Anderson et al., 2009). For sensitive or bloating-prone individuals, begin with 5–10 g/day of soluble, low-FODMAP fibers (psyllium or PHGG) and titrate upward over one to 2 weeks. Combining soluble and insoluble fractions within a balanced Mediterranean-style diet enhances stool form, microbial diversity, and SCFAs yield.

Several whole foods also show reproducible clinical benefits consistent with their fiber composition and fermentative potential:

- Two kiwifruits daily improve stool frequency and consistency, often outperforming psyllium (Gearry et al., 2023).
- Prunes (50–100 g/day) enhance bowel movements through the combined effects of sorbitol, polyphenols, and soluble fiber (Attaluri et al., 2011).
- Flaxseed and chia provide mucilaginous soluble fibers that increase stool hydration and facilitate transit (Soltanian and Janghorbani, 2018).
- Legumes, consumed two to three times weekly, supply fermentable fibers and prebiotic substrates that sustain microbial balance (Anderson et al., 2009).

To note, the British Dietetic Association Guidelines (Dimidi et al., 2025) confirmed that current evidence supports the use of specific foods or nutrients, particularly psyllium, kiwifruit, prunes, and magnesium-rich mineral waters, while data on whole-diet approaches remain insufficient.

Together, these foods exemplify how naturally occurring fibers can be incorporated into daily meals to reinforce physiological and microbial mechanisms of colonic motility. When dietary fiber alone provides incomplete relief, the addition of targeted prebiotics or probiotics may further support fermentation balance and propulsive activity, as discussed in the next section.

### 3.1 The microbiota-diet interface and microbial therapeutics

The intestinal microbiota plays a pivotal role in regulating gut motility, epithelial secretion, and visceral sensitivity (Wei et al., 2022). Dietary composition profoundly shapes microbial diversity and metabolic output, ultimately determining the balance between propulsive and inhibitory pathways of the colon.

Disruption of microbial ecology, due to antibiotics, low fiber intake, or ultra-processed foods, impairs SCFAs and bile-acid metabolism, contributing to delayed transit and dysmotility. Large-scale epidemiological data support the link between pro-inflammatory dietary patterns and impaired colonic motility. In an analysis of 8272 U.S. adults, higher Dietary Inflammatory Index scores were independently associated with a greater likelihood of constipation after adjustment for age, sex, BMI, energy intake, and comorbidities (Lu et al., 2025). Gender and low physical activity amplified this effect, indicating that systemic and mucosal inflammation may influence bowel function through neuroimmune pathways.

On the other side, prebiotic fibers such as inulin, fructoligosaccharides (FOS), galactooligosaccharides (GOS), and partially hydrolyzed guar gum (PHGG) selectively stimulate *Bifidobacterium* and *Lactobacillus* species, increasing SCFAs production, particularly acetate, propionate, and butyrate (Cardona et al., 2013). Short-chain fatty acids activate FFAR2 and FFAR3 receptors expressed on enteroendocrine L cells and enteric neurons, stimulating GLP-1 and PYY secretion and thereby accelerating colonic transit (Chambers et al., 2018). Microbial metabolites, particularly SCFAs such as butyrate, enhance tryptophan hydroxylase-1 (TPH1) expression and serotonin biosynthesis in enterochromaffin cells, reinforcing peristaltic reflexes and gut-brain communication (Buey et al., 2023; Wei et al., 2022).

In parallel, polyphenol-rich foods, including fruits, extra virgin olive oil (EVOO), cocoa, and green tea, modulate gut microbiota composition by promoting SCFA-producing genera such as *Bifidobacterium, Faecalibacterium, and Roseburia*, while reducing potentially pro-inflammatory taxa (*Enterobacteriaceae*, *Clostridium* spp.). These changes contribute to lower luminal pH, enhanced epithelial barrier integrity, and reduced mucosal inflammation (Cardona et al., 2013; Cuciniello et al., 2023) and they are amplified within a Mediterranean-style dietary pattern, which supports microbial diversity and resilience. Natural food sources include onions, garlic, asparagus, leeks, oats, and bananas, while PHGG represents a low-FODMAP, well-tolerated option for fermentation-sensitive patients (Gibson and Shepherd, 2010). Probiotics show moderate but reproducible benefits, reducing colonic transit time and improving stool form in meta-analyses (Dimidi et al., 2014; Waller et al., 2011).

The best-supported strains include *Bifidobacterium lactis HN019 and *Lactobacillus* casei Shirota*, typically administered at ≥10^9^ CFU/day for 4–8 weeks (Dimidi et al., 2014; Waller et al., 2011). Combining probiotics with fermentable fiber sustains colonization and SCFAs production. Symbiotic formulations, pairing compatible probiotic-prebiotic combinations, yield additive effects on stool frequency, consistency, and symptom tolerance (Marco et al., 2017). Otherwise, the British Dietetic Association Guidelines shows data on probiotics and synbiotics heterogeneous and strain-dependent, precluding firm conclusions (Dimidi et al., 2025). Fermented foods such as yogurt, kefir, tempeh, sauerkraut, and kimchi function as natural symbiotics, providing both live microorganisms and fermentable substrates that favor the growth of beneficial taxa. Controlled trials and translational studies show that regular consumption of fermented foods enhances gut microbial diversity, increases the abundance of SCFA-producing genera (*Bifidobacterium, Faecalibacterium, Roseburia*), and promotes cross-feeding networks that sustain acetate, propionate, and butyrate production (Marco et al., 2017).

Therapeutic outcomes, however, depend on strain specificity, baseline microbial composition, and constipation phenotype, reinforcing the need for personalized dietary integration rather than uniform recommendations (Wei et al., 2022). To note, adequate hydration is essential for the bulking effect of dietary fiber. Adults should aim for 1.5–2 L of total fluids daily, adjusted for body size, climate, and physical activity. Magnesium- and sulfate-rich mineral waters exert both osmotic and choleretic effects, promoting bile flow, water retention, and peristaltic activity. Effective regimens typically include 0.5–1 L/day of water containing at least 100 mg/L magnesium and 1,000 mg/L sulfate, preferably consumed before breakfast or between meals (Bothe et al., 2017). Improvements in stool frequency and consistency often occur within 1–2 weeks of regular consumption. In a classic crossover trial, high water intake (≥2 L/day) significantly enhanced the bulking effect of dietary fiber, increasing stool frequency and softness compared with low water intake (Anti et al., 1998). In contrast, trials in patients with CC indicate that water alone yields inconsistent improvements unless combined with soluble fiber supplementation or magnesium- and sulfate-rich mineral waters. In a double-blind randomized study, 1 L/day of Heilbronner magnesium-sulfate mineral water for 6 weeks significantly increased weekly bowel movements (+2.5 BM/week vs. +0.4 in placebo, p < 0.01) and improved Bristol Stool Scale scores without adverse effects (Bothe et al., 2017).

Furthermore, the laxative efficacy of such mineral waters reflects a dual osmotic-choleretic action, promoting bile flow, colonic secretion, and propulsive motility, effects particularly relevant in slow-transit constipation or bile-acid deficiency phenotypes (Vijayvargiya et al., 2018). A large glass of water on waking can trigger postprandial motility via vagal pathways (Rao et al., 2000), while sustained intake throughout the day maintains colonic responsiveness. Observational studies confirm that low total fluid intake, particularly when fiber intake is suboptimal, correlates with higher odds of constipation (Markland et al., 2013).

In contrast to SCFA-producing pathways, methanogenic microbiota has been associated with slowed colonic transit. Methane production correlates with delayed transit and constipation severity, and its reduction has been shown to accelerate colonic transit in a small, randomized pilot study, supporting a contributory, albeit not definitive, role of methanogenesis in constipation pathophysiology (Ghoshal et al., 2018).

### 3.2 Macronutrients and enteroendocrine hormones

Beyond overall food intake, individual macronutrients exert distinct and well-characterized effects on gastrointestinal motility through endocrine, neural, and bile-acid–mediated pathways. Dietary carbohydrates, particularly fermentable substrates, influence colonic motor activity indirectly via microbial metabolism and SCFAs production. Soluble, viscous fibers improve stool hydration and consistency and enhance propulsive activity, whereas excessive intake of rapidly fermentable carbohydrates may exacerbate bloating and discomfort, especially in IBS-C phenotypes (Müller-Lissner et al., 2005; Yang et al., 2012).

Dietary lipids represent potent modulators of postprandial motility. Fat ingestion stimulates CCK release, gallbladder contraction, and BA secretion, coordinating digestive processes while exerting segment-specific effects on motility. Although lipids slow gastric emptying and small-intestinal transit, bile acids reaching the colon activate secretory and propulsive mechanisms through TGR5 signalling, supporting colonic motility (Poole et al., 2010; Vijayvargiya et al., 2018). Very low-fat diets may therefore reduce bile-dependent motor stimulation, particularly in slow-transit constipation.

Proteins influence motility primarily through enteroendocrine signalling, including gastrin, CCK, and PYY release, contributing to postprandial motor coordination. While protein-induced effects on colonic transit are less pronounced than those of fiber BA, balanced protein intake supports physiological reflex activation without excessive fermentative burden (Beglinger and Degen, 2006; Drucker, 2006).

Among macronutrients, dietary fiber remains the most extensively studied component in CC. Clinical trials consistently show that soluble fibers, particularly psyllium, improve stool frequency and consistency, whereas insoluble fibers may worsen symptoms in selected patients due to increased gas production and luminal distension (Attaluri et al., 2011; Müller-Lissner et al., 2005).

At this stage, these mechanisms should be viewed as individual pieces of a broader physiological puzzle, many of which remain only partially defined. Clarifying how these hormonal, neural, and nutrient-driven signals integrate *in vivo* will be essential to translate this knowledge into phenotype-specific nutritional strategies rather than uniform macronutrient prescriptions.

### 3.3 Choleretic and sensory foods

Dietary modulation of BA signalling represents a promising, though still underexplored, avenue for restoring intestinal motility. Physiologically, BAs play a dual regulatory role. Activation of the FXR-FGF19 axis in the ileum provides negative feedback that slows bile synthesis and colonic secretion, thereby reducing transit. In contrast, stimulation of TGR5 receptors on enteric neurons and epithelial cells enhances chloride and water secretion, increases mucosal hydration, and promotes propulsive motor activity (Lefebvre et al., 2009; Poole et al., 2010).

Accordingly, nutritional strategies that favor TGR5 activation, by increasing bile flow or providing natural agonists, may therefore counteract hypomotility in slow-transit constipation or in patients with reduced choleretic tone. This rationale is supported by translational and physiological evidence linking bile acid availability to colonic secretion and motility, particularly in constipation phenotypes associated with reduced fecal bile acids (Vijayvargiya et al., 2018; Wahlström et al., 2016).

Bitter vegetables such as chicory, dandelion, radicchio, and artichoke contain sesquiterpene lactones that stimulate bile secretion and activate intestinal TAS2R bitter-taste receptors, generating a combined sensory and hormonal signal that triggers CCK release and colonic reflex activity (Depoortere, 2014; Chen et al., 2006). These foods operate at the intersection of sensory, endocrine, and biliary pathways and appear particularly suitable for slow-transit forms of constipation.

Dietary fats, particularly those rich in monounsaturated and polyunsaturated fatty acids, such as EVOO, nuts, and avocado, further enhance CCK-mediated gallbladder contraction and bile secretion. Beyond their choleretic action, such fats exert anti-inflammatory effects and support enteroendocrine signalling, reinforcing their role within balanced dietary patterns (Jiménez-Sánchez et al., 2022).

Hydration acts as an additional modulator of choleretic tone. Magnesium- and sulfate-rich mineral waters stimulate bile flow and provide osmotic laxative effects, improving stool frequency and consistency within one to 2 weeks of regular consumption (Bothe et al., 2017; Anderson et al., 2009). This combined osmotic-choleretic mechanism supports both colonic secretion and propulsive motility, particularly in slow-transit phenotypes.

### 3.4 When single nutrients act within an integrated meal

When examined in isolation, each nutrient, fiber, fat, protein, or bitter compound, acts on a specific physiological pathway. Meal composition itself modulates the gastrocolic reflex. In a controlled scintigraphic crossover study in healthy volunteers, high-fat meals produced the most pronounced colonic motor response compared with isocaloric carbohydrate or protein meals. This enhanced postprandial propulsion, associated with slower gastric emptying, was attributed to lipid-induced cholecystokinin release and vagal activation, a mechanism that couples nutrient composition with reflex motor patterns of the distal gut (Rao et al., 2000). Manometric studies confirmed that meal composition influences colonic motor activity: high-fat meals induce a slower-onset but more sustained colonic response, whereas high-carbohydrate meals produce a shorter, more propulsive pattern (Müller-Lissner et al., 2005).

Yet, within a real meal, these components interact dynamically, generating additive or modulatory effects on motility, bile flow, and microbial fermentation. Balanced meals that combine fermentable fibers, healthy fats, polyphenols, and natural bitters can simultaneously engage mechanical, microbial, and neurohormonal pathways, creating a coordinated stimulus for propulsion and secretion.

A Mediterranean diet (MD), rich in vegetables, fruits, legumes, whole grains, nuts, and olive oil, provides a nutrient-dense and microbiota-supportive framework that promotes regular bowel function (Jiménez-Sánchez et al., 2022). Conversely, diets high in red meat, refined carbohydrates, and ultra-processed foods are associated with slower transit and reduced microbial diversity.

In a controlled crossover trial, daily supplementation with 25 mL of EVOO for 3 weeks shortened total colonic transit, increased bowel frequency, and raised fecal bile-acid and SCFA concentrations, together with greater *Bacteroides* and *Faecalibacterium* abundance (Jiménez-Sánchez et al., 2022). These findings support that monounsaturated-fat-induced bile release and microbial modulation contribute to the mild choleretic and pro-motility effects of EVOO.

Similarly, in a randomized trial of 108 IBS patients, a Mediterranean low-FODMAP diet improved symptoms and maintained relief at 6 months, with fecal metabolomic shifts indicating reduced total and branched-chain fatty acids, consistent with a microbiota-metabolite mechanism sustaining clinical improvement (Gibson and Shepherd, 2010).

A meal with bitter vegetables such as chicory, radicchio, dandelion, and artichoke stimulates bile secretion and activates intestinal TAS2R receptors, eliciting a dual sensory–hormonal signal that enhances CCK release and colonic motility (Depoortere, 2014; Anderson et al., 2009). In selected patients, moderate inclusion of lightly fried foods prepared with fresh EVOO may further promote postprandial motility through coordinated CCK/PYY release, gallbladder contraction, and TGR5 activation (Poole et al., 2010; Anderson et al., 2009). This effect is most pronounced when consumed in the morning or after fasting, coinciding with peak vagal responsiveness. Conversely, repeated use of overheated oils or very low-fat diets can blunt choleretic and hormonal responses, predisposing to reduced bile flow and harder stool consistency.

### 3.5 Chrono-nutrition and meal timing

Meal timing exerts a major influence on GI motility by aligning nutrient intake with circadian fluctuations in enteric excitability, hormone release, and microbiota metabolism (Wahlström et al., 2016; Rao et al., 2000). Synchronizing eating patterns with these rhythms, known as chrono-nutrition, optimizes digestive efficiency and enhances colonic responsiveness.

The morning period represents the physiological peak of vagal tone and colonic motor sensitivity. Consuming a balanced breakfast within 30–60 min of waking activates the gastrocolic reflex through coordinated secretion of CCK, PYY, and motilin, promoting colonic contractions and stool passage (Rao et al., 2000; Voigt et al., 2019). Moderate inclusion of monounsaturated fats (e.g., EVOO, nuts, avocado) at breakfast may potentiates CCK/PYY-driven motility. In contrast, similar meal composition late in the evening may blunt motor responses because of reduced vagal tone and circadian downregulation of colonic activity (Voigt et al., 2019).

Coffee ingestion, whether caffeinated or decaffeinated, transiently increases colonic motor activity within minutes, likely through stimulation of the gastrocolic reflex rather than caffeine itself (Brown et al., 1990). Warm fluids may act as a nonspecific vagal stimulus, but evidence for an independent pro-motility effect is limited. Thus, the common practice of morning coffee or warm beverage intake may serve as a practical behavioral cue that coincides with peak colonic responsiveness rather than a true pharmacological trigger.

Spacing meals evenly throughout the day sustains postprandial colonic reflexes and promotes rhythmic motor activity, whereas irregular eating patterns, prolonged fasting, or late-night meals may disrupt circadian alignment, reduce vagal tone, delay gastric emptying, and attenuate colonic motor activity (Zarrinpar et al., 2016; Scheer et al., 2009).

**FIGURE 1:**
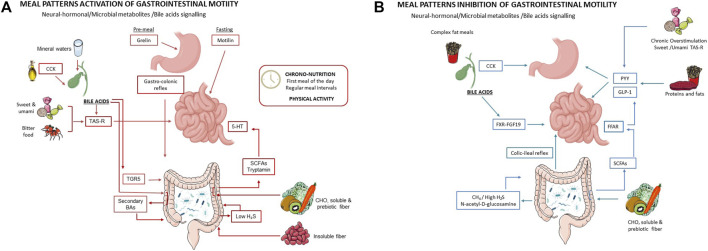
**(A)** Meal-related activation pathways of gastrointestinal motility. The panel illustrates key neural, hormonal, luminal, and microbial mechanisms coordinating gastrointestinal motility across the stomach, small intestine, gallbladder-biliary axis, and colon in relation to meal timing. During the pre-prandial and fasting phases, ghrelin primes gastric and small-intestinal motor activity, while motilin drives the migrating motor complex, promoting interdigestive propulsion. Meal ingestion activates vagal pathways and the gastro-colonic reflex, enhancing colonic motor responses. Luminal chemosensory signaling via bitter and umami taste receptors (TAS-R) modulates hormone secretion and enteric neural activity. Cholecystokinin (CCK), released in response to mono- and polyunsaturated fats, delays gastric emptying but stimulates gallbladder contraction and bile release, indirectly promoting distal intestinal and colonic motility. Bile acids then act through TGR5 receptors on epithelial and enteric neurons, enhancing chloride secretion, smooth-muscle relaxation, colonic propulsion, and neuromodulatory signaling. These effects are time-dependent and most pronounced in the morning, when vagal tone and CCK sensitivity are highest. Enterochromaffin cell–derived serotonin (5-HT), microbiota-derived short-chain fatty acids (SCFAs), and tryptamine produced from carbohydrate and fiber fermentation synergistically reinforce propulsive and secretory activity, particularly in the colon. Finally, soluble fibers and magnesium-sulfate–rich mineral waters support luminal hydration and coordinated intestinal transit. **(B)** Meal-related inhibition pathways of gastrointestinal motility. The panel depicts how specific dietary patterns, luminal signals, and circadian factors inhibit gastrointestinal propulsion. High-fat and protein-rich meals stimulate cholecystokinin (CCK) release, slowing gastric emptying and reducing downstream transit. Increased bile acid signaling activates the FXR-FGF19 axis, providing negative feedback on bile acid synthesis and gallbladder emptying, thereby limiting TGR5-mediated pro-motility effects. Chronic overstimulation of sweet and umami taste receptors (TAS-R) promotes inhibitory signaling within enteric and endocrine pathways. Excessive fermentation of soluble fibers may lead to excessive production of microbial metabolites, including short-chain fatty acids (via FFAR signaling) and gases (e.g., CH_4_, H_2_S), triggering inhibitory reflexes such as the colic-ileal reflex, resulting in luminal distension, delayed transit, and bloating. These inhibitory effects are accentuated after late-evening meals, when vagal tone and circadian responsiveness of gastrointestinal motility are reduced.

To conclude the section on the physiological basis of diet–motility modulation, Figures 1A,B illustrate the bidirectional effects of meals on intestinal motility, highlighting stimulatory pathways (Figure 1A) and inhibitory pathways (Figure 1B).

### 3.6 Translational and phenotype-oriented strategies

Personalized nutrition is crucial in the management of CC, as patient response varies according to motility phenotype, microbial profile, and tolerance to fermentable substrates (Dimidi et al., 2025; Buey et al., 2023; Müller-Lissner et al., 2005).

In slow-transit constipation, the primary goal is to stimulate propulsive activity and restore reflex responsiveness. A structured regimen providing 25–30 g/day of total fiber, with a balanced combination of soluble (e.g., psyllium, β-glucans) and insoluble fractions (e.g., wheat bran), improves stool bulk and frequency when introduced gradually and supported by adequate hydration (≥1.5–2 L/day) (Anderson et al., 2009; Yang et al., 2012).

While soluble fibers enhance fermentation and SCFA-driven neuromuscular signalling, insoluble fiber contributes mechanical distension that promotes colonic propulsion, particularly in individuals with preserved reflex activity.

Adjunctive dietary components may further support motility through sensory and biliary mechanisms. Including bitter vegetables and moderate healthy fats (EVOO, nuts) can enhance CCK-mediated gallbladder contraction and bile secretion, thereby providing a physiological stimulus for postprandial reflex activation (Depoortere, 2014; Jiménez-Sánchez et al., 2022). Although direct clinical evidence remains limited, increased bile availability may secondarily favour bile-acid–dependent pro-secretory and pro-motility signalling pathways, including TGR5-mediated mechanisms, as suggested by translational studies (Lefebvre et al., 2009; Poole et al., 2010; Vijayvargiya et al., 2018).

Although dietary fiber remains the cornerstone of nutritional therapy in CC, its universal efficacy has been questioned. Müller-Lissner et al. emphasized that CC is not uniformly fiber-deficient, and that excessive or poorly tolerated fiber may aggravate symptoms in subsets of patients, Müller-Lissner et al., (2005). This underscores the need for phenotype-based tailoring of fiber type and dose rather than uniform application across all forms of constipation.

In IBS-C or methane-positive CC, excessive fermentation may aggravate bloating and discomfort. A low-FODMAP adaptation emphasizing low-fermentable soluble fibers (psyllium or PHGG) is preferable (Gibson and Shepherd, 2010). Methane-associated slowing of transit may be mitigated by magnesium-rich mineral waters and moderate caffeine intake (Pimentel et al., 2006; Zarrinpar et al., 2016).

In post-antibiotic, the priority is microbiota restoration. Daily consumption of fermented foods (yogurt, kefir, tempeh) and prebiotic fibers (inulin, resistant starch) may support SCFAs production and related benefits on epithelial function and motility (Yang et al., 2012; Anderson et al., 2009; Marco et al., 2017).

A subgroup of CC patients, may show altered bile-acid metabolism. About 15% of IBS-C subjects present decreased fecal BAs correlating with slower transit (Vijayvargiya et al., 2018). These findings justify bile-stimulating dietary components, such as healthy fats and bitter vegetables (chicory, radicchio, artichoke), as potential adjuncts within targeted nutritional frameworks (Depoortere, 2014; Lefebvre et al., 2009; Poole et al., 2010; Jiménez-Sánchez et al., 2022).

In patients with dyssinergic constipation, dietary management plays only an ancillary role. The main goal of nutrition is to soften stool consistency and reduce straining, not to correct the underlying neuromuscular dysfunction. Studies have clearly demonstrated that biofeedback therapy (BFT) is significantly more effective than dietary modification, laxatives, diazepam, muscle relaxants, placebo, or sham biofeedback, with greater long-term efficacy and no adverse effects (Sadeghi et al., 2023).

Finally, diet may also be modulated according to colonic tone (Bassotti et al., 2005; Ravi et al., 2010; Cuomo and Habib, 2007). In hypertonic phenotypes (segmental contractions, painful evacuation, pellet-like stools), vagal-calming nutrients, warm liquids, soluble fibers, and olive-oil-based preparations may gently activate CCK/PYY pathways, reducing spasm and promoting coordinated propulsion. Conversely, atonic or hypomotile phenotypes (sluggish transit, reduced reflex sensitivity) may benefit from stimulating inputs, including bitter, lipid, or fermentative foods, which enhance choleretic signalling and reawaken propulsive reflexes.

Table 1 provides a summary of the practical dietary applications of CC.

**TABLE 1:** Practical dietary strategies for chronic constipation and corresponding levels of evidence.

Dietary target	Food or strategy	Practical recommendation	Level of evidence	Key references
Hydration	High mineral-content water	0.5–1 L/day of mg/SO_4_-rich water before breakfast or lunch	Clinical evidence (RCTs in CC)	Bothe et al., 2017; Anti et al., 1998; Dimidi et al., 2025
Soluble fiber optimization	Psyllium, inulin, oats (β-glucans) kiwi (soluble-rich mixed fiber)	Fiber target: 25–30 g/day; gradual increase over 1–2 weeks	Clinical evidence (multiple RCTs in CC)	Yang et al., 2012; Gearry et al., 2023; Attaluri et al., 2011; Dimidi et al., 2025
Foods with combined laxative mechanisms	Prunes (mixed fiber + sorbitol + polyphenols)	50–100 g/day; adjust to tolerance and bloating	Clinical evidence (RCTs in CC)	Attaluri et al., 2011; Yang et al., 2012; Dimidi et al., 2025
Insoluble fiber caution	Wheat bran, vegetable skins	Limit if bloating or IBS-C; use blended approach	Clinical and physiological evidence	Badiali et al., 1995; Anderson et al., 2009; Müller-Lissner et al., 2005
Bile flow enhancement	EVOO, bitter greens, moderate healthy fats	Regular inclusion at main meals	Physiological/translational evidence (bile flow and TGR5)	Poole et al., 2010; Vijayvargiya et al., 2018; Jiménez-Sánchez et al., 2022
Sensory-hormonal stimulation (taste receptors)	Bitter and umami-rich foods (chicory, radicchio, artichoke, citrus zest, fermented sauces; light sauté in EVOO)	Small portions daily, esp. At lunch; supports bile flow and post-meal motility via TAS2R/TAS1R	Physiological/Hypothesis-generating	Depoortere, 2014; Daly et al., 2013; Chen et al., 2006; Janssen et al., 2011
Fermented and prebiotic foods	Kefir, yogurt, tempeh; garlic, leeks, asparagus; PHGG	3-5×/week; use low-FODMAP sources if bloating	Translational and clinical evidence (small RCTs, pilots)	Marco et al., 2017; Dimidi et al., 2017; Buey et al., 2023; Waller et al., 2011
Chrono-nutrition	Breakfast within 1 h of waking; early dinner	Align meals with circadian motility peaks	Physiological/translational (healthy-subject motility and hormonal studies)	Voigt et al., 2019; Scheer et al., 2009; Rao et al., 2000; Zarrinpar et al., 2016
Fried/sautéed foods	Eggs or vegetables lightly fried in EVOO (fresh oil, moderate heat)	Morning use to trigger CCK/PYY and bile flow	Physiological/Hypothesis-generating (CCK-PYY-bile reflexes)	Tack et al., 2006; Depoortere, 2014
Personalization	By phenotype (slow-transit, IBS-C, reduced choleretic tone)	Adjust fiber fermentability, fat quality, fluids	Conceptual/Hypothesis-generating (needs phenotype-stratified trials)	Staudacher et al., 2014; Vijayvargiya et al., 2018
Physical activity	Daily walking, yoga, cycling ≥30 min	Enhances parasympathetic tone and reflex motility	Clinical/observational (meta-analyses and cohorts)	Gao et al., 2019; Dukas et al., 2003

## 4 Integrated dietary protocol proposal: the “SMART constipation diet”

The extent to which nutritional strategies can be used alone or in combination with pharmacological therapy depends on the severity of the disorder and the underlying motility phenotype. Coordinated modulation of multiple physiological pathways, neurohormonal, microbial, choleretic, and reflex, can enhance clinical outcomes when dietary interventions are personalized to emphasize the most relevant mechanism in each patient. Although clinical evidence remains limited, converging translational data support the feasibility of this multidimensional approach (Anderson et al., 2009; Wei et al., 2022; Buey et al., 2023; Jiménez-Sánchez et al., 2022; Dimidi et al., 2025).

Within this rationale, an integrated, evidence-based nutritional model, the SMART (Sensory, Motor, bile Acid and Reflex Tailored) Constipation Diet is proposed. The SMART constipation diet integrates mechanistic insights into a structured dietary framework that aligns nutrient composition, meal timing, and fermentation management with GBA (Camilleri, 2019; Gearry et al., 2023; Buey et al., 2023; Dimidi et al., 2025; Grundy and Schemann, 2006). However, as these components have largely been studied in isolation, at present it should be regarded as a conceptual, hypothesis-generating tool rather than a validated dietary treatment for CC, pending confirmation in adequately powered RTCs.

The SCD is built on five synergistic pillars: fiber optimization, hydration and mineral balance, fat quality and bile stimulation, microbiota support, and chrono-nutritional timing.

- Morning: Begin with 250–300 mL of warm or magnesium-sulfate mineral water upon waking to trigger the gastrocolic reflex. A substantial breakfast combining soluble fiber (e.g., oatmeal, kiwi, or prunes) with moderate fat and warm fluids enhances colonic motility (Gearry et al., 2023; Attaluri et al., 2011; Anderson et al., 2009). A teaspoon of EVOO or lightly fried components, such as eggs or vegetables sautéed in oil, provides an early choleretic stimulus (Depoortere, 2014; Jiménez-Sánchez et al., 2022). Light frying in EVOO, when performed with fresh oil and moderate heat, physiologically engages the gastrocolic reflex via lipid-mediated mechanisms (Rao et al., 2000; Jiménez-Sánchez et al., 2022).
- Midday: Three times per week, lunch should feature whole grains, legumes, and vegetables dressed in EVOO to sustain postprandial fermentation and SCFAs production (Wei et al., 2022; Anderson et al., 2009; Marco et al., 2017). Including bitter vegetables such as chicory, radicchio, dandelion, or artichoke enhances bile flow and supports colonic motility (Daly et al., 2013; Depoortere, 2014; Poole et al., 2010). Mildly bitter or umami-rich components (e.g., EVOO-based sautés, herbs, citrus zest, fermented sauces) engage intestinal taste receptors and enhance post-meal motility.
- Evening: A lighter dinner emphasizing cooked vegetables, fish or eggs, and low-FODMAP carbohydrates (e.g., rice or quinoa) supports overnight rest and avoids circadian misalignment (Voigt et al., 2019; Zarrinpar et al., 2016). Late-night meals should be avoided to preserve MMC function and microbiota synchrony (Voigt et al., 2019; Rao et al., 2000).
- Hydration: Maintain 1.5–2 L/day of total fluids, including 0.5–1 L of magnesium- or sulfate-rich mineral water before breakfast or lunch to enhance both osmotic hydration and bile flow (Bothe et al., 2017; Dimidi et al., 2025; Anti et al., 1998).
- Adjuncts: Include fermented foods (kefir, yogurt, tempeh) 3–5 times per week and prebiotic-rich vegetables (onions, leeks, asparagus) to promote microbial diversity and resilience (Marco et al., 2017; Wei et al., 2022; Cuciniello et al., 2023). Regular moderate physical activity (≥30 min/day) synergizes with dietary interventions by enhancing parasympathetic tone and propulsive reflexes (Gao et al., 2019; Brown et al., 1990).

## 5 Conclusions and future perspectives

Dietary modulation remains a cornerstone in the management of CC, underpinned by robust mechanistic evidence linking nutrients, hormones, BAs, and the gut-brain-microbiota axis. However, clinical validation beyond fiber remains limited, most studies on prebiotics, probiotics, and bile-acid-modulating foods are constrained by small sample sizes, heterogeneous designs, and narrow endpoints focused mainly on stool frequency.

A critical unmet need is the phenotype-specific evaluation of dietary interventions. Patients with slow-transit CC, IBS-C, methane-positive profiles, or defecatory disorders differ substantially in neurohormonal, microbial, and reflex regulation, yet are typically analyzed together, masking differential responses.

Within this evolving context, the SMART constipation diet represents a translational, hypothesis-generating framework, not a validated therapeutic protocol. It integrates five physiological pillars, fiber optimization, hydration and mineral balance, fat quality and bile stimulation, microbiota support, and chrono-nutritional timing, into a structured dietary model that can be personalized by severity, phenotype, and concomitant pharmacologic therapy. Overall, formal validation through randomized, phenotype-stratified clinical trials is required to determine its clinical efficacy, safety, and applicability across different constipation subtypes.
